# Chloroform in Obstruction of the Bowels from Spasms

**Published:** 1853-11

**Authors:** J. D. Cain


*Chloroform in Obstruction of the Bowels from Spasms.* By J. D. Cain, M.D.—Every physician meets, in the course of his practice, with cases of obstruction of the intestines, which has come gradually or suddenly, generally from some cause of irritation existing in them. The obstruction in these cases consists of a spasmodic contraction of a portion, or of portions of the intestines, generally the small. The plan I formerly pursued was, to cease all attempts at forcing a passage by means of cathartics, if one or two brisk cathartics failed, and to resort to opium freely, enemata of warm water, melted lard or butter, sweet oil, etc., the warm bath, fomentations of the abdomen, and other means of producing relaxation. For more than two years I have used chloroform, as a more powerful agent than opium, and its preparations, and as more certain in relaxing the muscular system. The chloroform administered in greater or less inhalation, soon produces a greater or less degree of resolution, and taking advantage of the relaxation thus effected, I give enemata, either stimulating, mucilaginous, or oily, which in a short time bring away fæcal matter. The inhalation may be repeated as often as in the judgment of the physician the case demands.

Chloroform possesses the immense advantage over opium, of relieving effectually and promptly the pain, and in not leaving the bowels in a constricted state, the sedative effect soon passing off.

Seven cases have thus been treated by me with highly satisfactory results. In one case only have I experienced any difficulty in inducing the requisite degree of relaxation of the bowels. The subject of this case was very slightly suspectible to its influence; but the pain was completely relieved by frequent inhalations, and the obstruction gradually overcome.—*Southern Med. and Surg. Journal.*

